# Early Epidemiological Assessment of the Virulence of Emerging Infectious Diseases: A Case Study of an Influenza Pandemic

**DOI:** 10.1371/journal.pone.0006852

**Published:** 2009-08-31

**Authors:** Hiroshi Nishiura, Don Klinkenberg, Mick Roberts, Johan A. P. Heesterbeek

**Affiliations:** 1 Theoretical Epidemiology, University of Utrecht, Utrecht, The Netherlands; 2 Centre for Mathematical Biology, Institute of Information and Mathematical Sciences, Massey University, Auckland, New Zealand; Virginia Tech, United States of America

## Abstract

**Background:**

The case fatality ratio (CFR), the ratio of deaths from an infectious disease to the number of cases, provides an assessment of virulence. Calculation of the ratio of the cumulative number of deaths to cases during the course of an epidemic tends to result in a biased CFR. The present study develops a simple method to obtain an unbiased estimate of confirmed CFR (cCFR), using only the confirmed cases as the denominator, at an early stage of epidemic, even when there have been only a few deaths.

**Methodology/Principal Findings:**

Our method adjusts the biased cCFR by a factor of underestimation which is informed by the time from symptom onset to death. We first examine the approach by analyzing an outbreak of severe acute respiratory syndrome in Hong Kong (2003) with known unbiased cCFR estimate, and then investigate published epidemiological datasets of novel swine-origin influenza A (H1N1) virus infection in the USA and Canada (2009). Because observation of a few deaths alone does not permit estimating the distribution of the time from onset to death, the uncertainty is addressed by means of sensitivity analysis. The maximum likelihood estimate of the unbiased cCFR for influenza may lie in the range of 0.16–4.48% within the assumed parameter space for a factor of underestimation. The estimates for influenza suggest that the virulence is comparable to the early estimate in Mexico. Even when there have been no deaths, our model permits estimating a conservative upper bound of the cCFR.

**Conclusions:**

Although one has to keep in mind that the cCFR for an entire population is vulnerable to its variations among sub-populations and underdiagnosis, our method is useful for assessing virulence at the early stage of an epidemic and for informing policy makers and the public.

## Introduction

When an emerging influenza virus appears in humans, an early concern is whether the virus has the potential to cause a devastating pandemic, i.e., the global spread of an infection killing a substantial number of people. To assess the pandemic potential, two critical aspects need to be studied: the transmission potential and the clinical severity of the infection [1]–[3]. It is widely known in epidemiology that the former aspect, the transmission potential, can be quantified by the reproduction number, i.e., the average number of secondary cases generated by a single primary case [1], [4], by characterizing the heterogeneous patterns of transmission (e.g. age-specificity) [5], and by measuring other epidemiological quantities such as household secondary attack rate. There are two different approaches to assessing the latter aspect of a pandemic, the virulence of infection. One is to explore specific genetic markers of the virus that are known to be associated with severe influenza (e.g. the PB1 gene) [6], although the absence of a known marker, as was for example the case in a novel swine-origin influenza A (H1N1) virus (S-OIV), does not necessarily indicate that the virus is benign [7]. Another is an epidemiological approach to quantification of the case fatality ratio (CFR), the conditional probability of death given infection (or disease; see below).

The CFR in general is vaguely defined as the ratio of deaths to cases, whose denominator should ideally be the total number of infections, but is frequently taken to be only the diagnosed cases due to the impossibility of counting all infected individuals. Because in the early phase of an outbreak information is often limited to confirmed cases, we concentrate on confirmed cases only, and refer to the CFR as the confirmed CFR (cCFR) for clarity. As the world has experienced a global spread of S-OIV since April 2009, methods have been sought for the real-time assessment of virulence by measuring the cCFR which is a representative of the epidemiological measurements of virulence [2], [3]. Nevertheless, a much-used crude estimate of the cCFR, i.e. the ratio of the cumulative number of deaths to cases at calendar time *t*, tends to yield a biased (and mostly underestimated) cCFR due to the time-delay from onset to death [8]; similar estimates of such a biased cCFR for severe acute respiratory syndrome (SARS) have shown how such estimates can vary substantially as an epidemic progresses, stabilizing only in the later stages of the outbreak [8], [9]. In the following we will use the terms biased and unbiased cCFR when we refer to this particular source of bias.

Improving an early epidemiological assessment of an unbiased cCFR is therefore crucial for the initial determination of virulence, shaping the level and choices of public health intervention, and providing advice to the general public [10]. To obtain an estimate of the cCFR, the lesson from the SARS outbreak is that a statistical technique is required that corrects the underestimation, e.g. a technique addressing censoring [8], [11], [12]. Nevertheless, in the case of novel S-OIV, an early unbiased estimation of the cCFR has appeared particularly challenging. Initial reports from the government of Mexico suggested a virulent infection, whereas in other countries the same virus was perceived as mild [13]. In the USA and Canada there were no deaths attributed to the virus in the first 10 days following a declaration of a public health emergency by the World Health Organization. Even under similar circumstances at the early stage of the global pandemic, public health officials, policy makers and the general public want to know the virulence of an emerging infectious agent. That is, a simple method for assessing cCFR is called for, even when only a few deaths have been reported, or even when there has been no report of deaths. Except for another unbiased cCFR estimate in Mexico (0.4%, range 0.3–1.5%) [1], this early assessment has been missing. In the USA, a technical discussion has taken place on the crude measurement of the biased cCFR using the cumulative numbers of deaths and confirmed cases so far [10].

In line with this, an epidemiological method and its practical guide for early assessment of virulence are called for. The present study aims at developing a simple method to assess the virulence of an emerging influenza virus at the early stage of the epidemic, even when there have been only a few deaths or none at all. The method takes into account the time from the onset of symptoms to death, while differing from previously published statistical methods which employ censoring techniques [8], [11]. As an example, we give an early prediction of the cCFR of S-OIV infection in the USA and Canada, and show that the unbiased cCFR, as estimated by our method at the early stage of the epidemic in these countries, was in fact comparable to that estimated for Mexico [1]. Our unbiased estimation of the cCFR does not address all sources of error in data (e.g. underdiagnosis of infected individuals) and we summarize the relevant issues in the discussion.

## Materials and Methods

### Theoretical background

We assess the virulence of S-OIV by measuring the risk of death, expressed as the cCFR. The cCFR is interpreted as the conditional probability of death given confirmed diagnosis [14]. Since the data of S-OIV infection we use in the present study are only confirmed cases, we have replaced “infection” in the denominator of CFR by confirmed diagnosis of infection (see Discussion). Accordingly, an unbiased estimator of cCFR would be the proportion of deaths among confirmed cases at the end of an epidemic. Although one could instead assess the virulence by measuring the proportion of hospitalized cases among a total number of confirmed cases, criteria for hospital admission are not universal, being influenced by isolation policies and in some regions by cultural and social differences.

In the following, the notation used to represent the three different statistical measurements of cCFR is: (i) *b*_t_, which is a crude, biased estimate of the cCFR calculated at time *t*; (ii) *π*, which is an unbiased cCFR to be estimated in the present study, and is the unknown parameter that governed the outbreaks; and (iii) *p*_t_, a random variable, which yields an estimator of *π* (see below) and is regarded as the realized value in one particular outbreak. First, *b*_t_, a crude and biased estimate of cCFR, calculated at time *t*, is given by the ratio of the cumulative number of deaths *D*_t_ to the cumulative number of confirmed cases *C*_t_:
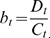
(1)During the outbreak of severe acute respiratory syndrome (SARS) in 2002–03, it was shown that this estimator, *b*_t_, considerably underestimates the cCFR [8]. This is easily demonstrated by relating *C*_t_ and *D*_t_ to the incidence function *c*_t_ (i.e. the number of new confirmed cases on day *t*), and the conditional probability density function *f*_s_ of the time from onset to death, given death. First, *C*_t_ is the cumulative number of confirmed cases up to time *t*:
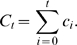
(2)Second, *D*_t_ is the cumulative number of deaths up to time *t*:
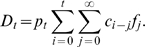
(3)As we mentioned above, *p*_t_ is the realized proportion of confirmed cases to die from the infection, and is a random variable, which would be an unbiased estimator for *π*. Therefore, *b*_t_ can be rewritten as
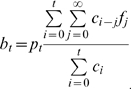
(4)As can be observed in equation (4), the estimator *b*_t_ is smaller than the realized *p*_t_, because the time delay from onset to death, expressed in the double summation in the numerator, results in the numerator being smaller than the denominator (note that *f*_s_ is a probability distribution). Therefore we refer to *b*_t_ as the biased estimator of the cCFR: it gives a biased estimate, calculated on day *t*, of the cCFR [8], [11]. When we observe the entire course of an epidemic (i.e. *t*→∞), *b*_t_ tends to *p*_t_ and becomes an unbiased estimator. The aim is to obtain an unbiased estimator “well before” observing the entire course of the outbreak.

### Statistical estimation

An adjustment of the estimator *b*_t_ by a factor of underestimation is achieved by rearranging equation (4):
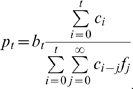
(5)We use *p*_t_ as the unbiased estimator of *π*, which is informed by three pieces of information: the cumulative number of deaths *D*_t_; the incidence *c*_t_; and the distribution of the time from onset to death *f*_s_. The former two are observed during the course of an epidemic. When there are a few deaths or none at all, an assumption has to be made for *f*_s_, e.g. from literature based on previous outbreaks (see below for detailed descriptions of *f*_s_). We call the multiplicative factor in equation (4) the factor of underestimation, *u*_t_, defined by
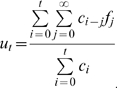
(6)The estimator *p*_t_ can be written as *p*_t_ = *b*_t_/*u*_t_.

**Figure 1:**
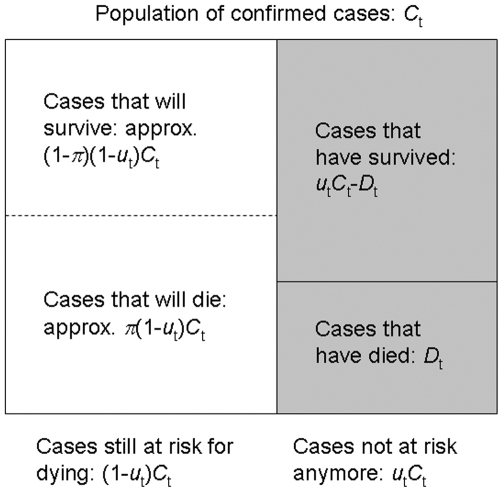
The population and sampling process for estimating the unbiased confirmed case fatality ratio during the course of an outbreak. At time *t* we know the cumulative number of confirmed cases and deaths, *C*_t_ and *D*_t_, and wish to estimate the unbiased case fatality ratio *π*, by way of the factor of underestimation *u*_t_. If we knew *u*_t_ we could specify the size of the population no longer at risk (*u*_t_ *C*_t_, shaded), although we do not know which surviving individuals belong to this group. A proportion *π* of those in the group still at risk (size (1- *u*_t_)*C*_t_, unshaded) is expected to die. Because each case no longer at risk had an independent probability of dying, *π*, the number of deaths, *D*_t_, is a sample from a binomial distribution with *n* = *u*_t_ *C*_t_, and *p*_t_ = *π*.

Figure 1 depicts the concept of the sampling scheme. The cumulative number of cases *C*_t_ is regarded as the total population size. Of these, only a proportion *u*_t_ has been at risk for dying by time *t*, whereas the outcome for the remaining proportion 1 - *u*_t_ is still unobserved. Among the *u*_t_ *C*_t_ cases that have been at risk, *D*_t_ have died and *u*_t_ *C*_t_ – *D*_t_ have survived the infection. This is a sample from a binomial distribution with sample size *u*_t_ *C*_t_ and probability *π*:
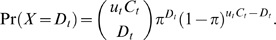
(7)An alternative way of deriving this probability is by first considering the total number, *y*, of people in the sample *C*_t_ that will ultimately die from infection, which is binomially distributed with sample size *n* = *C*_t_ and probability *π*. However, because of the time delay from onset to death, we do not observe this outcome by time *t*: only for a proportion *u*_t_ is the outcome observed. Hence our observation is a hypergeometric sample from a population of size *C*_t_, with sample size *u*_t_ *C*_t_, and number of deaths *y* [15], [16]:
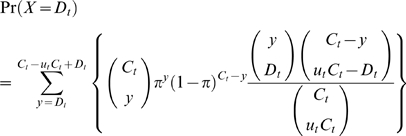
(8)which is equivalent to equation (7). We can use equation (7) as a likelihood function to obtain the maximum likelihood estimate of *p*_t_:
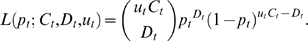
(9)The 95% confidence interval of *p*_t_ is derived from the profile likelihood. Further technical details, especially where an exponential growth of incidence is observed, are given in the Supporting Information S1.

### Quantitative illustrations

For calculation of the factor of underestimation *u*_t_, two pieces of information are needed: the incidence function *c*_t_ and the distribution of time from onset to death *f*_s_. For *c*_t_, we use the published dates of onset among confirmed cases, while *f*_s_ is assumed known.

**Figure 2:**
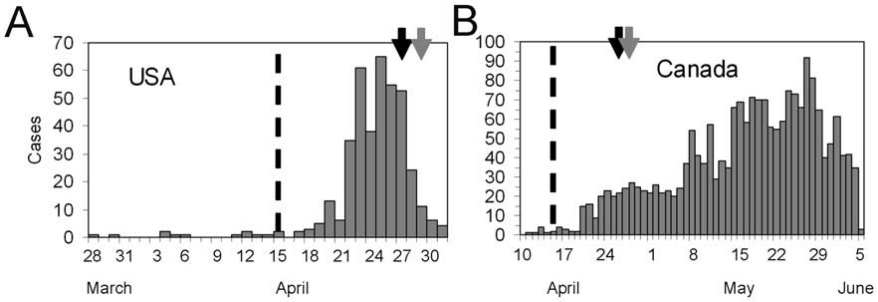
Temporal distribution of the date of onset for an H1N1 influenza epidemic in the USA and Canada, 2009. Epidemic curves of confirmed cases of human infection with swine-origin influenza A (H1N1) virus (S-OIV) with known date of onset in (A) the USA (n = 394) and (B) Canada (n = 2004). The vertical dashed line is the date on which the Centers for Disease Control and Prevention identified S-OIV. The World Health Organization increased the pandemic alert level from 3 to 4 on April 27 (black arrow) and then to 5 on April 29 (gray arrow). It should be noted that confirmed cases include substantial numbers of imported cases from abroad. In Canada, a few cases whose dates of onset were unable to be traced are also included according to their dates when a specimen was collected (the exact number of such cases is not known). Assuming that their impact on our estimation procedure is negligibly small, we regard all cases in B as representing the dates of onset.

We analyze empirical datasets of two different infectious diseases: SARS in Hong Kong (2003) and S-OIV infection in the USA and Canada (2009). First, we examine a simplified version of our method by using only deaths and cases from an early stage of the SARS epidemic, and compare our estimate against the eventual stable estimate at the end of the epidemic. For simplicity, we employ an exponential distribution for the distribution of the time from onset to death, *F*(s), with a mean of 35.9 days [11], and *f*_s_ is subsequently calculated as the daily increase in *F*(*s*), i.e., *f*_s_ = *F*(*s*)−*F*(*s*−1). Second, we use the most recent published datasets of S-OIV epidemics in which the dates of illness onset for confirmed cases are known [17], [18]. The latest such reports for the USA and Canada were at May 1 and June 10, 2009, respectively. In the USA, there were 399 confirmed cases by May 1, with 394 known dates of onset (Figure 2A). Among 399 confirmed cases, 2 cases resulted in death by May 1. In Canada, there were 2978 confirmed cases, with 2004 known dates of onset by June 10, among which 4 cases died by June 10 (Figure 2B). The biased cCFR estimates, *b*_t_ in these countries were 0.50% ( = 2/399) and 0.13% ( = 4/2978), respectively. The six deaths are insufficient to determine the distribution of time from onset to death for these countries. We therefore employ a gamma distribution for *F*(*s*) (to calculate *f*_s_), with reference to historical data for H1N1 [19], with a mean length of 9 days and a variance of 39.7 days^2^ (coefficient of variation 70%, shape parameter 2.04) [20]. To address the uncertainty, we examine the sensitivity of our unbiased cCFR estimate to different means (6–14 days) and variances (9–159 days^2^). See Supporting Information S2 for further technical details.

For the unbiased cCFR, we use 399 and 2978 cases, respectively, as our *C*_t_ in equation (9) for the USA and Canada. Similarly, *D*_t_ is 2 and 4 deaths, respectively. Nevertheless, since the adjustment of underestimation requires dates of symptom onset, we use 394 and 2004 cases for computing *u*_t_. Although this has little impact on the estimate for the USA, the cCFR in Canada is likely to be underestimated by our estimator, because the majority of the 974 cases whose dates of onset have yet to be clarified, may have experienced their symptom onset close to the latest time point of observation. We subsequently compare cCFR estimates between the USA and Canada by means of Fisher's exact test. For the hypothesis testing, the number of deaths, *D*_t_, as well as the number of those survived, calculated as *u*_t_ *C*_t_−*D*_t_, is compared between two countries.

## Results

### SARS: the case of exponential growth phase

The factor of underestimation *u* during the exponential growth phase is independent of time *t* and given by
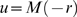
(10)where *M*(-*r*) is the moment generating-function of *f*(*s*), given the exponential growth rate *r* which is estimated via a pure birth process (see Supporting Information S3). That is, when *f*(*s*) is the density of an exponential distribution with mean *T*, we have *u* = M(−*r*) = 1/(1+*rT*).

**Figure 3:**
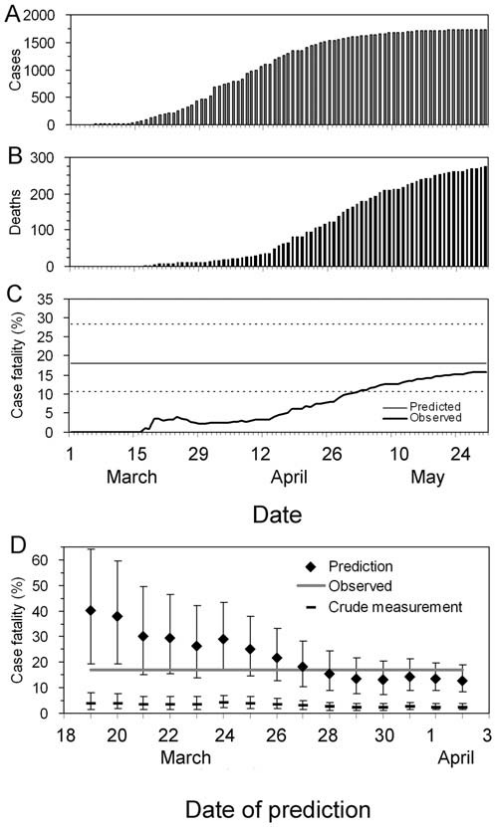
Early determination of the unbiased confirmed case fatality ratio of severe acute respiratory syndrome (SARS) in Hong Kong, 2003. (A & B) Cumulative numbers of confirmed cases and deaths. The increase in death is delayed in observation because of the time delay from onset to death. (C) Observed biased confirmed case fatality ratio (cCFR) estimates as a function of time (thick line) calculated as the ratio of the cumulative number of confirmed cases to deaths at time *t*. The estimate at the end of an outbreak (i.e. 302/1755 = 17.2 %) is the realized cCFR by the end of the epidemic. The horizontal continuous line and dotted lines show the expected value and the 95% confidence intervals of the predicted unbiased cCFR estimate (based on our method) only by using the observed data until 27 Mar 2003 (estimated at 18.1 % (95% CI: 10.5, 28.1). The 95% confidence interval was derived from profile likelihood. (D) The comparisons between the realized cCFR (horizontal grey line), the unbiased cCFRs based on observations by calendar time *t*, and the biased cCFR estimates, *b*_t_, given by the ratio of deaths to cases. Each prediction was obtained by using the exponential growth rate *r* up to time *t* and the cumulative numbers of deaths and cases at time *t*, and the mean time from onset-to-death of 35.9 days [11] which is assumed to follow an exponential distribution. Overestimation is seen in the early stages of the epidemic, but the 95% confidence limits in the later stages include the realized cCFR.

Figures 3A and 3B show the cumulative numbers of cases and deaths of SARS, and Figure 3C the observed (biased) cCFR estimates as a function of time, i.e. the ratio of the cumulative number of cases to deaths at time *t*. Due to the delay from onset of symptoms to death, the biased estimate of cCFR at time *t* underestimates the realized cCFR at the end of an outbreak (i.e. 302/1755 = 17.2 %). Nevertheless, even by only using the observed data for the period 19 March to 2 April, equation (10) yields an appropriate prediction (Figure 3D), e.g. the unbiased cCFR at 27 Mar is 18.1 % (95% CI: 10.5, 28.1). An overestimation is seen in the very early stages of the epidemic, but the 95% confidence limits in the later stages include the realized cCFR (i.e. 17.2 %).

### Influenza (H1N1) in 2009: the case of a few deaths

**Figure 4:**
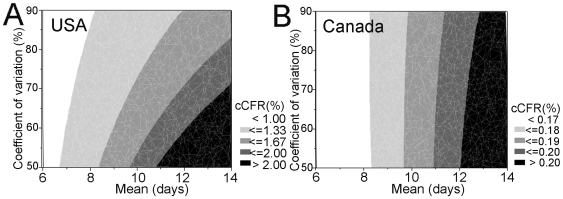
Sensitivity of the unbiased confirmed case fatality ratio of an influenza virus (H1N1) infection to different means and coefficients of variation of the time from onset to death in the USA and Canada, 2009. The contours show the maximum likelihood estimate of the unbiased confirmed case fatality ratio as a function of the mean and coefficient of variation of the time from onset-to-death in (A) the USA and (B) Canada. The estimates are based on observation by May 1 and June 10, respectively, with 2 and 4 deaths among a total of 399 and 2978 confirmed cases, respectively. A gamma distribution is employed for the time from onset to death, *f*(*s*). Both the quantitative and qualitative patterns of the USA differ from those of Canada, because the epidemic curve in the USA include more cases who developed the disease recently than those in Canada. It should be noted that the contour gray scales are different in (A) and (B).

When only a few deaths have been reported at the early stage of an epidemic, the unbiased cCFR estimate is given by minimizing the negative logarithm of the likelihood (see equation (9)). Given 2 and 4 deaths in the USA and Canada, respectively, and employing a gamma-distributed time from onset-to-death, the unbiased estimates of the cCFR are 1.23% (95% confidence interval (CI): 0.21, 3.76 %) and 0.18% (95% CI: 0.05, 0.41%) in the USA and Canada, respectively. The estimate in the USA appears significantly higher than that in Canada (Fisher's exact test; p<0.01). The uncertainty bounds on the unbiased cCFR estimates in both countries overlap with that estimated for Mexico [1]. Sensitivity analysis suggests that the expected values may lie in the range of 0.81–4.48% and 0.16–0.22% in the USA and Canada, respectively (Figure 4).

### Influenza (H1N1): the case of no death

Even when there has been no observation of death by time *t*, it would be useful for policy makers to understand the implication of no deaths for interpreting virulence in a conservative way. When *D*_t_ = 0 equation (7) simplifies to:
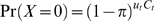
(11)which would result in an unbiased cCFR estimate of 0. Because sampling a finite number of cases during the course of an outbreak cannot prove that infection never results in death, a more useful result would be the maximum cCFR with a certain level of confidence if no deaths are observed after *C*_t_ cases. To obtain this result, we rearrange equation (11) to obtain
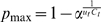
(12)where *p*_max_ is the maximum cCFR given *C*_t_ cases and no deaths, at a confidence level of 1-*α*, e.g. 95% if *α* = 0.05. Equation (12) is useful for obtaining a conservative estimate of virulence (i.e. upper bound of possible cCFR estimates) when no deaths have been reported by time *t*. In particular, during the early exponential growth phase the factor of underestimation, *u*, is independent of *t*.

**Figure 5:**
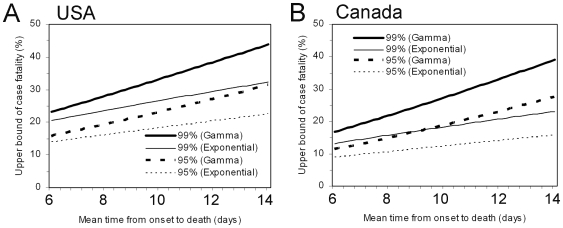
Upper bound of the confirmed case fatality ratio when there is no report of death. Upper bound of the cCFR (confirmed case fatality ratio) estimates in (A) the USA and (B) Canada, given no deaths by April 21 and April 24, 2009, respectively (based on 42 and 91 cases). The upper bounds are examined for significance levels at 95% and 99% to find at least 1 death. Gamma and exponential distributions were employed to model the distribution of time from onset to death.

Assuming that the exponential growth phase of influenza continued until April 21 and 24, 2009, respectively, in the USA and Canada, *r* in these countries is estimated at 0.183 (95% CI: 0.133, 0.245) per day and 0.300 (95% CI: 0.241, 0.367) per day, respectively (see Supporting Information S3). The resulting *p*_max_ in the USA and Canada (based on 42 and 91 cases and no deaths) is shown in Figure 5. These upper bounds are examined for confidence levels at 95% and 99%. If the mean and variance of the time from onset to death are 9 days and 39.7 days^2^, and we employ a gamma distribution, *p*_max_ is estimated at 21.2% and 30.7% at *α* = 0.05 and 0.01 in the USA. Similarly, *p*_max_ in Canada is estimated at 16.8% and 24.6% at *α* = 0.05 and 0.01, respectively.

## Discussion

We propose a new epidemiological method for assessing the virulence of an emerging infectious disease at the early stage of an epidemic. The results with the Hong Kong SARS dataset prove the usefulness of this method that corrects the biased cCFR estimator which is simply the ratio of cumulative deaths to cases. Early in the epidemic, the ultimately realized cCFR is within the confidence interval obtained by our method. The proposed method is particularly useful when an epidemic curve of confirmed cases is the only data available (i.e. when individual data from onset to death are not available, especially, during the early stage of the epidemic).

Our estimates suggest that the virulence of S-OIV H1N1 infection is comparable to the virulence observed in past influenza pandemics of the 20th century (<2.0 % for the 1918–19 pandemic and<0.5 % for the 1957–58 pandemic [21]). Although our estimates may not be as high as 2.0%, and even though the unbiased cCFR estimate for the USA is a likely overestimation (see below), we should emphasize that antiviral treatment and other medical interventions have been instituted from the beginning of this pandemic. Our results show that the few observations of death in the USA and Canada give us no reason to believe that the unbiased cCFR, and therefore the virulence of the novel pandemic strain, is smaller in the USA and Canada than in Mexico. Nevertheless, given that the CFR of seasonal influenza is equal to or less than 0.1% [10], our estimates (with the lower bound of cCFR close to the 0.1%) do not offer conclusive results to indicate that the S-OIV is more virulent than seasonal influenza, but do point in that direction.

It should be noted that our method only adjusts underestimation due to time delay from onset to death, and other epidemiological characteristics associated with unbiased estimation of the cCFR have yet to be addressed. In the present study, we estimated the cCFR as the proportion of deaths among confirmed cases. This definition was chosen, because of our aim to use the minimally available data, and so we were not able to estimate the proportion of deaths among all symptomatic cases, and not able to estimate the proportion of deaths among all those infected (symptomatic and asymptomatic). The issue of defining the correct denominator population can never be completely resolved, but it is essential to realize how the obtained estimate relates to other situations [8]. By only using confirmed cases, it is clear that all cases will be missed that do not seek medical treatment or are not notified, as well as all cases that are asymptomatic. This means that our cCFR estimate is higher than the proportion of deaths among infecteds, and may be considered an overestimate. However, when relating our estimate to previous pandemics, it should also be realized that the current pandemic is the first where many confirmatory diagnoses of influenza have been recorded using RT-PCR techniques, allowing improved precision of cCFR estimates over those for previous influenza epidemics. Whereas the use of RT-PCR in the current pandemic may yield a smaller denominator (and thus an overestimate of CFR compared to previous pandemics), other pandemics could have involved substantial numbers of false-positive cases in the denominator. Developing a method which permits comparable assessment of virulence is ongoing.

**Figure 6:**
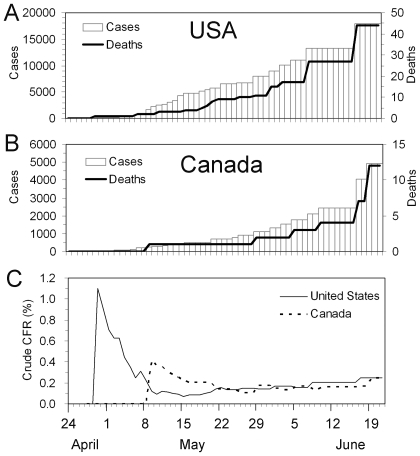
Time variations in the biased confirmed case fatality ratio of an H1N1 influenza epidemic in the USA and Canada, 2009. Cumulative numbers of confirmed cases and deaths in (A) the USA and (B) Canada. Cases (bars) and deaths (thick lines) are comparatively shown. (C) The biased estimates of confirmed case fatality ratio (cCFR) given by the ratio of deaths per confirmed cases. The data were extracted from irregular situation updates of the World Health Organization [25], and the horizontal axis (time) corresponds to the date of reporting. Therefore, it should be noted that the estimate suffers reporting delay, and in this sense, the calculated biased cCFR is different from our *b*_t_ (based on date of onset) in the main text. The most recent report was made on June 19. Since the interval of update has been irregular, the cumulative number of cases and deaths is kept the same as the latest report when there was no update on the corresponding date.

Figure 6 shows the time course of biased cCFR estimates in the USA and Canada based on the reporting date of confirmed cases and deaths to the World Health Organization. Note that the estimates in Figure 6C are different from our *b*_t_ due to unavailability of the date of onset, although they give an approximate indication of the time-course of the biased cCFR. It is striking to see that the biased cCFR during the very early stage (i.e. from late April to mid-May) showed a declining trend following a single spike. The biased cCFR estimates at later time points show a slight increase as a function of time, which is consistent with our knowledge of underestimation of the cCFR [8]. The early spike may be explained by a time-varying coverage of confirmed diagnoses which could have increased as a function of time (i.e. cases in the very beginning of the epidemic were less likely to be confirmed). Other plausible explanations include (1) demographic stochasticity, (2) effective treatment, and (3) heterogeneous risk of death among subpopulations. As for (1), because the number of deaths in the USA and Canada was very small during the early stage, the spike may reflect (unpredictable) probabilistic variations in the number of deaths among a small number of confirmed cases. If that is the case, our unbiased cCFR estimate for the USA (with data until May 1) may be too high, not because of a systematic bias but just by chance. In relation to factor (2), it is plausible that cases diagnosed in later stages of the epidemic receive treatment at an early stage of illness (or even before symptom onset). With respect to (3), the risk of dying is likely to be different for different subpopulations [8], [10], [22], [23]. It should be noted that the composition of sub-populations (e.g. age-groups and those with a specific underlying disease) is likely to vary as a function of time, and a cCFR estimate for the entire population, such as ours, is influenced by this variation. These points need to be addressed in future studies.

To fully clarify the virulence and its epidemiological characteristics (e.g. variable risks by age and underlying diseases), two lessons for surveillance and data sharing should be noted. First, rather than updating the data based on date of reporting, it is critically important to summarize the data according to the date of onset both at local and global levels. Knowing the date of symptom onset is a key to applying our proposed estimation framework to empirical observation. Second, epidemiological data should be updated in a precise reporting interval at least during the early stage of an epidemic (so that the data permit estimation of the unbiased cCFR). Given that mean time from onset to death is around 9 days, weekly data do not enable us to make our explicit adjustment. Optimal reporting for the early cCFR estimation may be incorporated into official pandemic response plans. Moreover, in addition to using death as an outcome of virulence, the usefulness of other epidemiological measurements of severe manifestation (e.g. the number of admissions to intensive care unit) needs to be explored.

Despite a need to further clarify heterogeneous risks of death for the S-OIV pandemic, early assessment of virulence by means of our unbiased cCFR estimator is useful for informing policy makers and the general public about the potential severity of an infectious disease (of course, one needs to ensure an understanding of the above mentioned bias among non-experts). We have shown that underestimation can be adjusted in a very simple manner, and our approach enabled us to obtain an unbiased cCFR estimate by only minimizing a binomial deviance. These methods are particularly useful when there have been only a few deaths or even no death at all by time *t* during the course of an epidemic. Uncertainties surrounding the unbiased estimate of cCFR based on a few deaths can partly be addressed by sensitivity analysis of the estimate to different lengths of time from onset to death. An observation of zero deaths in a given country (or a specific setting) should not be deemed a signature of a “benign” virus without observing a substantial number of cases. We have shown that a conservative upper bound of cCFR is a more useful interpretation of the observed number of cases without death. In this way, given that we have some prior knowledge or a few observations of death which permit us to assume *F*(*s*) is known, epidemiologists and biostatisticians in each country or locality can directly apply our method to assess the virulence of an infection at the early stage of any emerging infectious disease.

During the final stages of revision, it came to our attention that an epidemiological study on cCFR of S-OIV with similar techniques and statistical philosophy has been published online [24], indicating that the preliminary estimate of cCFR for a combination of the USA, Canada and Mexico is 0.5% and emphasizing a need to accurately capture the cases for the denominator.

## Supporting Information

Supporting Information S1 (0.05 MB DOC)
