# Supplementary material for: Early Epidemiological Assessment of the Virulence of Emerging Infectious Diseases: A Case Study of an Influenza Pandemic
Source: PLoS One. 2009 Aug 31;4(8):e6852. doi: 10.1371/journal.pone.0006852 (PMC2729920; doi:10.1371/journal.pone.0006852)
Supplement: Supporting Information S1 — (0.05 MB DOC) [file pone.0006852.s001.doc]

### **Supporting Information**

### **Title: Early epidemiological assessment of the virulence of emerging infectious diseases: a case study of an influenza pandemic**

## Hiroshi Nishiura, Don Klinkenberg, Mick Roberts, Johan AP Heesterbeek

**Technical details**

**S1. Approximations for the exponential growth phase**

Whereas we discussed incidence in discrete time in the main text, here we use continuous time arguments for mathematical convenience; incidence and factor of underestimation at time *t* are denoted by *c*(*t*) and *u*(*t*), respectively. Although the most precise estimates of the unbiased cCFR are obtained by calculation of *u*(*t*) by use of equation (6) (so that *p*_t_ can be estimated), we have also derived a simple approximation for *u*(*t*) in the early (exponential) growth phase of an epidemic. If we observe an exponential increase of onsets during the early stage of an epidemic with growth rate *r*, the expectation of incidence E(*c*(*t*)) can be written as

$$\mathrm{E}(c(t))=c_{0}\exp(rt)$$

where *c*_0_ is a constant. It should be noted that *r* may be different from the so-called intrinsic growth rate [S1], in that our incidence E(*c*(*t*)) includes not only cases caused by indigenous secondary transmissions but also imported cases. By use of equation (A1), equation (6) becomes

$$u=\frac{\int_{0}^{t} \exp\left(r\tau\right)\int_{0}^{\infty} \exp\left(-rs\right)f\left(s\right)dsd\tau}{\int_{0}^{t} \exp\left(r\tau\right)d\tau}=\int_{0}^{\infty} \exp(-rs)f(s)ds$$

which is independent of calendar time *t*. Equation (A2) indicates that the factor of underestimation *u* during the exponential growth phase is given by the moment generating-function of *f*(*s*), i.e. equation (10) in the main text, given the exponential growth rate *r*. That is, when *f*(*s*) is the density of an exponential distribution with mean *T*, we have *u* = M(-*r*) = 1/(1+*rT*). When *f*(*s*) is the density of a gamma distribution with mean *T* and coefficient of variation *v*, we have *u* = M(-*r*)= $\left(1+rTv^{2}\right)^{-\frac{1}{\nu^{2}}}$.

**S2. Historical data on the time from onset to death**

In addition to the observed number of deaths and cases, a piece of information needed to estimate the factor of underestimation *u*(*t*) is the distribution of the time from onset to death, *f*(*s*). For this we use evidence from an epidemic of H1N1 in 1918-19 [S2,S3], and from recent human infections with H5N1 [S4, S5], because well-informed distributions of S-OIV infection in the USA and Canada require many more observations of death. The median time from onset-to-death for H1N1 in 1918-19 ranged from 7 to 10 days (with mean of 8-9 days), and the times for H5N1 have been reported to range from 4-30 days. We employed two different distributions for the time from onset-to-death *f*(*s*), i.e. exponential and gamma distributions with a mean time of 9 days, and the gamma distribution with a variance of 39.7 days^2^ (coefficient of variation 70%, shape parameter 2.04) [S3]. We addressed the uncertainty with respect to *f*(*s*) by performing sensitivity analyses of the estimate *p* to different mean times ranging from 6 to 14 days, and to different coefficients of variation ranging from 50 to 90%.

During the 1918-19 pandemic (H1N1) the density *f*(*s*) was said to exhibit a bimodal pattern due to a secondary bacterial infection accounting for a second peak around 22-24 days since first symptom onset of influenza [S2,S3,S6]. Both exponential and gamma distributions are unimodal and do not capture this property. Nevertheless, we employed a unimodal distribution because (A) severe cases in the USA and Canada are likely to be treated with antibiotics which would prevent this second peak, and (B) the impact of variations in variance on equation (6) can be partially addressed by sensitivity analysis of the unbiased cCFR estimates to different means and variances (with gamma distribution).

**S3. Estimation of the exponential growth rate**

We employed exponential and gamma distributions for *f*(*s*) and thus *u* = 1/(1 + *rT*) and $\left(1+rTv^{2}\right)^{-\frac{1}{\nu^{2}}}$, respectively, to estimate the upper bound of the cCFR in the USA and Canada during the exponential growth phase, i.e. using equation (12) in the main text. The exponential growth was assumed to have continued until April 21 and April 24 2009, respectively. We estimated *r* from the observed incidence through a likelihood-based method. Since demographic stochasticity cannot be ignored during the early growth phase of an outbreak, a stochastic approach was taken. We estimate the exponential growth rate *r* based on the following pure birth process [S7]:

$$\begin{matrix} \Pr(c(t+h)=n\left|c(t)=n\right.)=1-rnh+o(h), \\ \Pr(c(t+h)=n+1\left|c(t)=n\right.)=rnh+o(h), \\ \Pr(c(t+h)\geq n+2\left|c(t)=n\right.)=o(h). \end{matrix}$$

The detailed process of finding an analytical solution of the model (A3) is given elsewhere [S7, S8]. Given our observations of the cumulative number of cases, *C*(0), *C*(1), *C*(2), …, *C*(*t*), we have

$$\Pr\left(C\left(t\right)=n+m\left|C\left(t-1\right)=n\right.\right)=\left(\begin{matrix} n+m-1 \\ n-1 \end{matrix}\right)\exp\left(-rn\right)\left(1-\exp\left(-r\right)\right)^{m}$$

which was used to construct a likelihood function for *r*,

$L\left(r\right)\propto\exp\left(-r\sum_{i=0}^{t-1} C\left(i\right)\right)\left(1-\exp\left(-r\right)\right)^{C\left(t\right)-C\left(0\right)}$.

Equation (A5) was used for estimating *r*. The 95% CIs were derived from profile likelihood.

**References**

S1. Diekmann O, Heesterbeek JAP (2000) Mathematical Epidemiology of Infectious Diseases: Model Building, Analysis and Interpretation. New York: Wiley.

S2. Brundage JF, Shanks GD (2008) Deaths from bacterial pneumonia during 1918-19 influenza pandemic. Emerg Infect Dis 14: 1193-1199.

S3. Nishiura H (2007) Time variations in the transmissibility of pandemic influenza in Prussia, Germany, from 1918-19. Theor Biol Med Model. 4: 20.

S4. Wong SS, Yuen KY (2006) Avian influenza virus infections in humans. Chest. 129: 156-68.

S5. Writing Committee of the Second World Health Organization Consultation on Clinical Aspects of Human Infection with Avian Influenza A (H5N1) Virus, Abdel-Ghafar AN, Chotpitayasunondh T, Gao Z, Hayden FG et al (2008) Update on avian influenza A (H5N1) virus infection in humans. N Engl J Med. 358: 261-273.

S6. Mills CE, Robins JM, Lipsitch M (2004) Transmissibility of 1918 pandemic influenza. Nature. 432: 904-906.

S7. Bailey NTJ (1964) The elements of stochastic processes with applications to the natural sciences. New York: Wiley.

S8. Karlin S, Taylor HM (1975) A First Course in Stochastic Processes (Second Edition). New York: Academic Press.
